# Targeted Next-Generation Sequencing Analysis for Recurrence in Early-Stage Lung Adenocarcinoma

**DOI:** 10.1245/s10434-020-09276-x

**Published:** 2020-11-02

**Authors:** In Ae Kim, Jae Young Hur, Hee Joung Kim, Jung Hoon Park, Jae Joon Hwang, Song Am Lee, Seung Eun Lee, Wan Seop Kim, Kye Young Lee

**Affiliations:** 1 Precision Medicine Lung Cancer Center, Konkuk University Medical Center, Seoul, Republic of Korea; 2 Department of Pulmonary Medicine, Konkuk University School of Medicine, Seoul, Republic of Korea; 3 Department of Pathology, Konkuk University School of Medicine, Seoul, Republic of Korea; 4 Macrogen Inc., Seoul, Republic of Korea; 5 Department of Thoracic Surgery, Konkuk University School of Medicine, Seoul, Republic of Korea

## Abstract

**Background:**

Despite surgical resection, early lung adenocarcinoma has a recurrence rate of 20–50%. No clear predictive markers for recurrence of early lung adenocarcinoma are available. Targeted next-generation sequencing (NGS) is rarely used to identify recurrence-related genes. We aimed to identify genetic alterations that can predict recurrence, by comparing the molecular profiles of patient groups with and without recurrence.

**Methods:**

Tissues from 230 patients with resected stage I–II lung adenocarcinoma (median follow-up: 49 months) were analyzed via targeted NGS for 207 cancer-related genes. The recurrence-free survival according to the number and type of mutation was estimated using the Kaplan–Meier method. Independent predictive biomarkers related to recurrence were identified using the Cox proportional hazards model.

**Results:**

Recurrence was observed in 64 patients (27.8%). In multivariate analysis adjusted for age, sex, smoking history, stage, surgical mode, and visceral pleural invasion, the CTNNB1 mutation and fusion genes (ALK, ROS1, RET) were negative prognostic factors for recurrence in early-stage lung adenocarcinoma (HR 4.47, *p* = 0.001; HR 2.73, *p* = 0.009). EGFR mutation was a favorable factor (HR 0.51, *p* = 0.016), but the CTNNB1/EGFR co-mutations were negative predictors (HR 19.2, *p* < 0.001). TP53 mutation was a negative predictor compared with EGFR mutation for recurrence (HR 5.24, *p* = 0.02). Conclusions: Targeted NGS can provide valuable information to predict recurrence and identify patients at high recurrence risk, facilitating selection of the treatment strategy among close monitoring and adjuvant-targeted therapy. Larger datasets are required to validate these findings.

**Electronic supplementary material:**

The online version of this article (10.1245/s10434-020-09276-x) contains supplementary material, which is available to authorized users.

Complete surgical resection with mediastinal lymph node dissection is the curative treatment for patients with early-stage lung cancer.^1^ However, 20–50% of these patients experience recurrence and eventually die of recurrent lung cancer (5-year overall survival, 58–73%).^2^,^3^ Thus, recurrence hampers the chances of complete cure after early resection of lung cancer. To prevent relapse, adjuvant chemotherapy is recommended for patients with stage II–III lung cancer. However, the benefit for stage IB patients is controversial, and it is not recommended for stage IA patients despite the high relapse risk. Adjuvant chemotherapy for stage IB is optional and recommended for patients according to pathologic risk factors such as visceral pleural invasion, poor differentiation, and size of the tumor without considering genetic mutations. Guidelines are needed for genetic risk stratification for stage I patients with worse than expected prognosis.

Genetic alterations are emerging as biomarkers with increasing importance for treatment selection and outcome of patients with non-small-cell lung cancer (NSCLC). Numerous biomarker studies have been conducted to predict recurrence after early resection using various platforms, such as gene expression profiling, quantitative reverse transcriptase-polymerase chain reaction (PCR), microRNA assays, mass spectroscopy, and next-generation sequencing (NGS).^4^ However, the genetic biomarkers for recurrence remain undetermined, and the molecular mechanism of tumor recurrence 1–2 years after complete resection is unknown.^3^ Mutations in KRAS,^5^ TP5^3^,^6^ and EGFR^7^ have been evaluated as genetic biomarkers for recurrence in early-stage lung cancer. However, the results have been inconsistent.^8^

The development of NGS has enabled the detection of multiple genetic alterations in a single assay. By using this approach, several co-occurring mutations in addition to driver mutations, which influence clinical outcomes, can be identified simultaneously. NGS studies have been performed to find the correlation between genetic alterations and clinical outcomes such as drug response, progression-free survival (PFS), and overall survival (OS) at advanced stages.^9^,^10^ However, only a few studies have attempted to describe recurrence-associated genomic alterations in early-stage NSCLC.^11^,^12^ In this study, we hypothesized that specific genetic alterations might affect recurrence. To identify genetic risk factors for recurrence, we compared the molecular profiles of patient groups with and without recurrence and attempted to reveal what genetic alterations are related to recurrence of resected early lung adenocarcinoma through targeted NGS analysis.

## Methods

### Patients and Sample Collection

The study cohort consisted of 230 patients who underwent surgery from September 2005 to May 2017 and were histologically confirmed with stage I–II lung adenocarcinoma according to the 8th American Joint Committee on Cancer (AJCC) criteria.^13^ The records were retrospectively reviewed. In addition to pathologic data, age, sex, smoking history, stage, surgical information (surgery date, methods and extent of resection, lymphatic and blood vessel invasion, and visceral-pleural invasion), and the dates of recurrence and death were collected. We excluded patients who concomitantly had another cancer and received any neoadjuvant treatment or radiotherapy. Patients underwent preoperative staging with CT, PET scans, and brain MRI. The clinical outcome data were collected until November 2018. The study protocol was approved by the Konkuk University Medical Center Institutional Review Board (approval number: KUH 1210049), and the need for written informed consent from the participants was waived due to the retrospective nature of this study and the lack of harm to patients.

### Next-Generation Sequencing Processing

DNA was extracted from formalin-fixed, paraffin-embedded tissues of 201 patients with pulmonary adenocarcinoma using the QIAamp DNA kit (Qiagen) according to the manufacturer’s protocol, and targeted NGS for 170 cancer-related genes and 37 fusion-related genes (KF1 panel, Supplementary Table S1) was performed using the Custom Cancer Panel v2.1 (Agilent Technologies, Inc., Santa Clara, CA, USA). Genomic DNA (200 ng) was fragmented using a Covaris E220 instrument (Covaris, Woburn, MA, USA), and subsequently subjected to end repair, tailing, and adapter ligation. Unligated adapters were removed with Agencourt AMPure XP beads (Beckman Coulter, Beverly, MA, USA). The resulting libraries were PCR-amplified and purified with Agencourt AMPure XP beads. Libraries were sequenced on an Illumina HiSgeq 2500 platform with an average sequencing depth of 1000 × . Unfortunately, matched germline DNA of patients as a normal control for mutation analysis was not available for this retrospective study.

### NGS Data Analysis

Raw sequencing data were processed, and variants were called using the Macrogen Inc. bioinformatics pipeline. The detailed process is described in Supplementary Method 1. Somatic mutations, including single nucleotide variants (SNVs), small insertions and deletions (Indels), and gene rearrangements and copy number variations (CNVs) were identified. Pathogenic somatic mutations whose variant allele frequencies (VAFs) were greater than 2% were regarded as significant actionable mutations and were used for analyses. Fusion genes were determined using the Ventana (D5F3) CDx assay or fluorescent in situ hybridization (FISH) for anaplastic lymphoma kinase (ALK), quantitative RT-PCR (Amoy) for ROS1, and FISH for RET rearrangement. Detailed FISH or RT-PCR methods are provided in Supplementary Method 2.

### Patient Follow-Up

We examined patients at 2-month intervals on an outpatient basis. The follow-up evaluation included physical examination, chest radiography, and blood examination, including chest CT scans. Whenever any symptoms or signs of tumor recurrence were detected, CT scans of the chest and abdomen, PET-CT, and brain MRI evaluations were performed. We diagnosed tumor recurrence based on physical examination and diagnostic imaging findings, which was confirmed histologically when feasible. Secondary primary lung cancer was differentiated from recurrent NSCLC according to the criteria proposed by Martini and Melamed.^14^ Date of recurrence was defined as the date of histologic proof or, in patients whose diagnoses were based on clinicopathological findings, the date of identification by the physician.

### Statistical Analysis

Clinical and pathological parameters of the patients were investigated by *χ*^2^ analysis or Fisher’s exact test (when appropriate) for categorical variables. Prognostic values were assessed by survival analysis. Recurrence-free survival (RFS) was defined as the time from surgery to recurrence or last follow-up in surviving patients. RFS percentages were calculated using the Kaplan–Meier method based on the genetic alteration subgroup, and the differences were tested by the log-rank test. The Cox proportional hazards model was used to test the effect of a mutation subtype adjusted for multiple clinical/pathological factors (sex, age, smoking status, stage, and surgical method). For all calculations, the tests were two-sided, and significance was set at 5%. Analyses were performed using the Statistical Package for the Social Sciences (SPSS) for Windows version 25 (SPSS, Inc., Chicago, IL, USA).

## Results

### Clinicopathological Characteristics of the Patients

The study included 230 patients with resected stage I–II lung adenocarcinoma at our institution from 2005 to 2017. Recurrence was observed in 64 of the 230 patients included in the study (27.8%). The median follow-up time was approximately 49 months. All patient characteristics are summarized in Table 1. Briefly, 48.3% of the patients were older than 65 years, 53.9% were male, and 49% were smokers. Most patients (82.2%) underwent radical lobectomies and 21% had visceral pleural invasion tumors. Patients with visceral-pleural invasion showed a significantly higher recurrence rate than patients without invasion (48.3% vs 20.5%, *p* < 0.001).

**Table 1:** Patients’ clinicopathological characteristics

Characteristics	All patients	Recurrence (%)	No recurrence (%)	*p* value
The number of patients	230	64 (27.8)	166 (72.2)	< **0.001**
Age	64.3 ± 11.5	62.3 ± 12.6	64.9 ± 10.9	0.12
< 65 years	119 (51.7)	38 (31.9)	81 (68.1)	0.22
≥ 65 years	111 (48.3)	26 (23.4)	85 (76.6)	
Sex				0.54
Male	124 (53.9)	34 (27.4)	90 (72.6)	
Female	106 (46.1)	30 (28.3)	76 (71.7)	
Smoking history				0.83
Non-smoker	116 (51.0)	33 (28.4)	83 (71.6)	
Ever-smoker	114 (49.0)	31 (27.2)	83 (72.8)	
Smoking dose	15.4 ± 20.2	17.0 ± 21.8	15.7 ± 19.5	0.67
Stage^a^				< **0.001**
IA(IA1 + IA2 + IA3)	141 (61.3)	27 (19.1)	114 (80.9)	
IB	61 (26.5)	23 (37.7)	38 (62.3)	
IIA	8 (3.5)	2 (25)	6 (75)	
IIB	20 (8.7)	12 (60)	8 (40)	
Surgical procedure				0.17
Sublobar resection	41 (17.8)	15(36.6)	26 (63.4)	
Lobectomy	189 (82.2)	49 (25.9)	140 (74.1)	
Tumor differential grade (*n* = 183)^b^				0.16
WD^c^	47 (25.7)	8 (17.0)	39 (83.0)	
MD^d^	115 (62.8)	31(27.0)	84 (73.0)	
PD^e^	21 (11.5)	8 (38.1)	13 (61.9)	
Pathologic invasion				
Visceral-pleural invasion	60 (21.0)	29 (48.3)	31 (51.7)	< **0.001**
Lympho-vascular invasion	14 (4.9)	9 (64.3)	5 (35.7)	**0.002**
Adjuvant chemotherapy	61 (26.5)	28 (45.9)	33 (54.1)	< **0.001**
Death	26 (11.3)	24 (92.3)	2 (7.7)	< **0.001**

Bold values denote statistical significance at the *p* < 0.05 level

^a^Pathologic stage was determined according to the American Joint Committee on Cancer (8^th^ edition)

^b^Histologic differentiations were available for 183 of 230 patients

^c^Lepidic type adenocarcinoma was classified as well differentiated (WD) tumor

^d^Acinar and papillary type adenocarcinomas were classified as moderately differentiated (MD) tumor

^e^Micropapillary and solid type adenocarcinomas were classified as poorly differentiated (PD) tumor

**Fig. 1:**
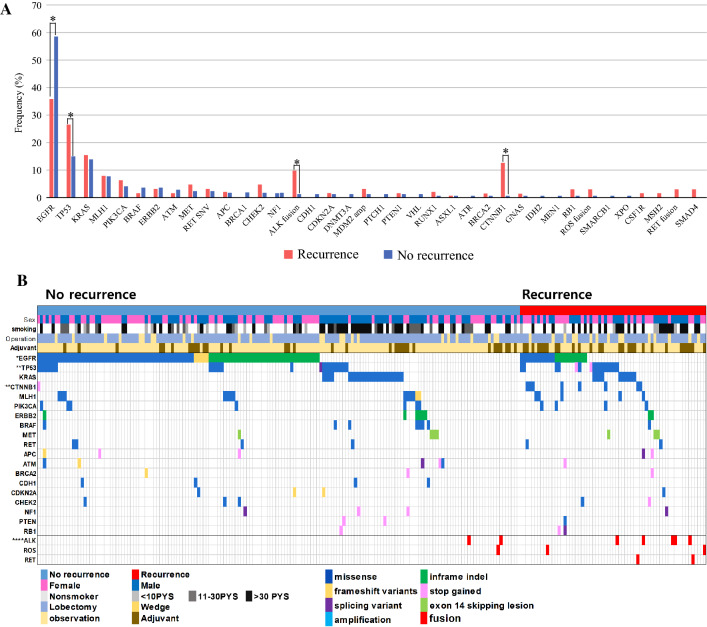
The genetic landscape in 230 early stage lung adenocarcinomas. **a** Comparison of the frequency of genetic alterations according to recurrence status. **b** Genetic landscape according to recurrence status. The *asterisk* indicates a statistical difference (*p < 0.05) of the genetic frequency in patients with recurrence and without recurrence. Alteration types are represented by the *colors* indicated. The frequency of patients with CTNNB1 mutation and fusion gene were statistically different between recurrence and no recurrence groups

EGFR mutations were the most frequently observed alteration (52.2%), followed by mutations in TP53 (18.3%) and KRAS (14.3%) (Supplementary Fig. 1). Next, we compared the frequency of genetic alterations according to recurrence status. Patients with no recurrence had more frequent EGFR mutations than patients with recurrence (58.4% vs 35.9%, *p* = 0.002), whereas patients with recurrence had a higher frequency of CTNNB1 mutation [12.5% (8/64) vs 0.6% (1/166), *p *< 0.001] and fusion genes [14.0% (9/64) vs 1.8% (3/166), *p* = 0.002] (Fig. 1a). Eight of 9 patients with CTNNB1 mutations and 9 of 12 patients with fusion genes experienced recurrence. Individual genetic alterations in the 230 patients with early-stage I–II lung adenocarcinoma are shown in Fig. 1b.

### Association Between the Number of Genetic Alterations and Clinical Factors for Recurrence

**Fig. 2:**
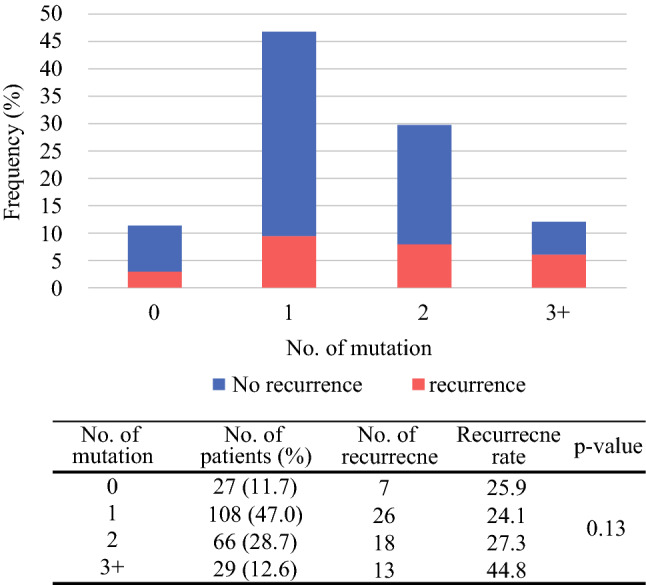
Recurrence rate according to the number of mutations. Distribution of number of mutations in 230 stage I–II lung adenocarcinomas

We investigated how the number of genetic alterations was distributed by performing targeted NGS. We observed that most patients (88.3%) had at least one pathogenic mutation. However, RFS showed no significant difference by the number of pathogenic alterations (Fig. 2, *p* = 0.13). Interestingly, patients without driver mutations (*n* = 23, 11.4%) showed short RFS similar to that of patients with multiple mutations (Supplementary Fig. 2). The number of genetic alterations did not correlate with age or smoking history either, unlike previous research results (data not shown). This difference might be due to the use of targeted NGS, rather than whole gene or whole exome sequencing, which might have revealed the association detected in previous research.

### Effect of Actionable Genetic Alterations and Clinicopathologic Factors on Recurrence in Early-Stage Lung Adenocarcinoma

**Fig. 3:**
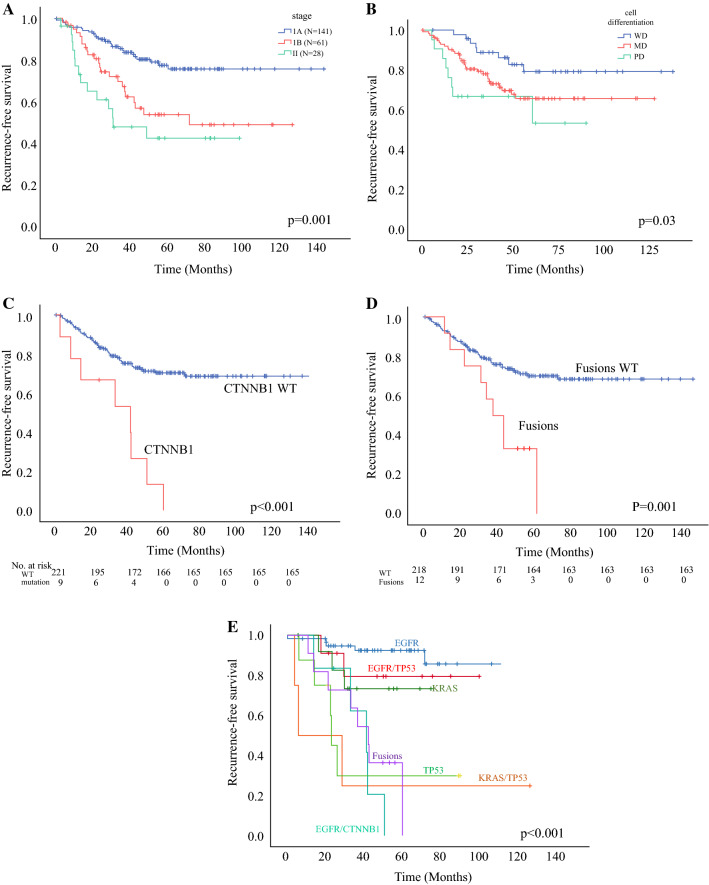
Effect of clinical-pathologic factor and genetic alterations on recurrence in early stage lung adenocarcinoma. **a** Kaplan–Meier curve comparing the RFS according to stage. **b** Kaplan–Meier curve comparing the RFS according to cell differentiation. RFS was related to histologic differentiation. We classified cancer tissues into 3 grades according to cell differentiation. Well differentiated (WD) cancers were lepidic type, moderate differentiated (MD) tissues were acinar type and papillary type, and the poorly differentiated (PD) group was micropapillary type and solid type. **c** Kaplan–Meier curve according to the CTNNB1 mutation status, **d** fusion genes (ALK, ROS1, and RET), and **e** combination subgroups of major driver mutation. This graph enabled us to estimate the possibility of recurrence according to co-occurring genetic alteration

We compared the RFS for all 230 patients according to clinical or pathological factors. Stage at diagnosis was significantly associated with RFS (Fig. 3a, HR 2.73, *p* = 0.001). Patients with poorly differentiated tumors also showed shorter RFS than patients with well or moderately differentiated tumors (HR 2.0, *p* = 0.03; Fig. 3b).

We compared RFS according to individual genetic alterations. Notably, CTNNB1 mutations were a significant poor prognostic marker for recurrence in univariate analysis (*p* < 0.001) (Fig. 3c). In multivariate analysis adjusted by age, sex, smoking history, stage, and surgery modality, the presence of CTNNB1 mutations was significantly associated with poor RFS (HR 4.47, CI 2.06–9.71, *p* = 0.001; Table 2). Patients with ALK, ROS1, or RET rearrangements were categorized as one group because the frequency of fusion genes was very low. The fusion genes (ALK, ROS1, and RET) were also independent prognostic genetic markers for recurrence in multivariate analysis (HR 2.73, 95% CI 1.28–5.86, *p* = 0.009; Fig. 3d, Table 2).

**Table 2:** Univariate and multivariate analysis of RFS in early stage lung adenocarcinoma according to genetic alterations

Category	Variables	Univariate analysis			Multivariate analysis		
		HR	95% CI	*p* value	HR	95% CI	*p* value
Age	≥ 65 versus < 65	0.79	0.48–1.31	0.36	0.93	0.55–1.57	0.81
Sex	Male versus female	0.95	0.58–1.55	0.84	0.57	0.24–1.36	0.2
Smoking history	Ever-smoker versus non-smoker	0.99	0.61–1.62	0.98	1.53	0.64–3.67	0.33
Pathologic stage^a^	II versus I	**2.73**	1.51–4.95	**0.003**	1.69	0.85–3.37	0.13
Grade of differentiation	PD versus WD/MD	2.02	0.94–4.33	**0.03**	**2.9**	**1.24**–**6.90**	**0.01**
Extension of surgery	Sublobar resection or lobectomy	1.4	0.71–2.78	0.32	1.44	0.74–2.80	0.27
Pathologic invasion	VPI versus none	**2.99**	**1.83**–**4.90**	< **0.001**	**2.63**	**1.44**–**7.81**	**0.018**
Adjuvant chemotherapy	Adjuvant chemotherapy	**2.55**	**1.55**–**4.19**	< **0.001**	**2.21**	**1.14**–**4.24**	**0.017**
EGFR mutation	All EGFR versus wild	**0.46**	**0.28**–**0.47**	**0.004**	**0.51**	**0.29**–**0.88**	**0.016**
	EGFR/TP53 versus EGFR	1.68	0.51–5.59	0.39	1.89	0.47–7.58	0.36
CTNNB1	CTNNB1 versus wild	**4.45**	**2.12**–**9.36**	< **0.001**	**4.47**	**2.06**–**9.71**	**0.001**
	EGFR/CTNNB1 versus EGFR	**6.61**	**2.60**–**16.8**	< **0.001**	**19.2**	**4.74**–**78.1**	< **0.001**
TP53 mutation	TP53 versus wild	**1.92**	**1.1**–**3.35**	**0.02**	**1.94**	**1.10**–**3.44**	**0.02**
	TP53 only versus EGFR only	**4.69**	**1.75**–**12.5**	**0.002**	**5.24**	**1.32**–**20.76**	**0.02**
KRAS mutation	KRAS versus wild	1.17	0.59–2.23	0.65	0.93	0.44–1.93	0.84
	KRAS only versus EGFR only	2.21	0.66–7.37	0.19	2.22	0.48–10.2	0.3
	KRAS/TP53 versus EGFR only	**5.68**	**1.84**–**17.5**	**0.002**	**11.5**	**2.00**–**66.2**	**0.006**
Fusion genes	ALK, ROS1, RET versus wild	**3.02**	**1.48**–**6.11**	**0.002**	**2.73**	**1.28**–**5.86**	**0.009**

*CI* Confidence intervals, *HR* Hazard ratio, *EGFR* Epidermal growth factor receptor, *VPI* Visceral-pleural invasion, *PD* Poor differentiation, *MD* Moderate differentiation, *WD* Well differentiated, *p* values were calculated using multivariate Cox proportional hazard models, adjusted for age, sex, smoking status, stage, and extension of surgery

^a^Pathologic stage was determined according to the American Joint Committee on Cancer (8th edition)

EGFR mutations were good prognostic factors for recurrence (HR 0.51, 95% CI 0.29–0.88, *p* = 0.016), and tumors with EGFR/TP53 dual mutations showed similar RFS to single EGFR mutations (HR 1.89, *p* = 0.36). In contrast, the EGFR/CTNNB1 double mutation showed a significantly shorter RFS in multivariate analysis. (HR 19.2, *p* < 0.001) (Fig. 3e, Table 2). Patients with a single TP53 alteration had shorter RFS than patients with a single EGFR mutation (HR 5.24, *p* = 0.02). Patients with KRAS/TP53 dual-mutation also experienced significantly shorter RFS than those with an EGFR mutation (HR 11.5, CI 2.0–66.2, *p* = 0.006) (Fig. 3e, Table 2).

### Individual Characteristics of Patients with CTNNB1 Mutations or Fusions

We investigated the clinical characteristics and patterns of recurrence in patients harboring CTNNB1 mutations or fusion genes. The prevalence of CTNNB1 mutations was low (3.9%, 9/230), but most of them (88.8%, 8/9) with these mutations experienced recurrence. All patients who experienced recurrence had a missense mutation of CTNNB1 in exon 3. The patient without recurrence despite the CTNNB1 mutation had a stop gain mutation in exon 4. Most cases with recurrence were distant metastasis (6/8; 2 cases in brain, 3 cases of multiple lung metastases, and 1 case of Lt adrenal). Therefore, the CTNNB1 mutations seem to be associated with distant metastases rather than local recurrence, and similar results have been reported in previous research^15^,^16^ (Table 3). Patients with CTNNB1/EGFR co-mutations showed a significantly shorter RFS than patients with a single EGFR mutation (Fig. 3c, *p* < 0.001). A single EGFR mutation is a good prognostic factor, but we should carefully examine the co-existence of CTNNB1 mutations with an EGFR mutation to predict recurrence. We also compared the RFS between the adjuvant therapy group and observation group in patients with the CTNNB1 mutation. There was no significant difference in RFS (*p* = 0.51, Supplementary Fig. 3). Fusion genes such as ALK, ROS1, or RET were rare mutations with a frequency of less than 5%, but most patients experienced recurrence. The recurrence pattern associated with fusion mutations was also distant metastases, for example, in the brain (Table 3).

**Table 3:** Individual characteristics of patients with CTNNB1 mutations or fusion genes

Case	Age	Sex	Site	Surgery	Stage	Adjuvant	EGFR mutation	Concurrent alterations	Recurrence	RFS (mon)	Recurrence site	CTNNB1 exon ID	CTNNB1 DNA change	VAF (%)	Characterization	Protein
*CTNNB1*																
1	71	F	RLL	Lobec.	1A2	N	21L858R	TP53, VHL	N	24.4	NA	4/15	c.283C > T	5.26	Stop gained	p.R95*
2	49	F	LUL	Lobec.	1A2	N	21L858R	T790M	R	50.9	LLL effusion	3/15	c.134C > T	29.8	Missense	p.S45F
3	68	M	RUL	Lobec.	1Bs^a^	Y	21L858R	JAK2,RUNX1	R	42.1	brain	3/15	c.122C > T	2.11	Missense	p.T41I
4	65	F	LUL	Lobec.	1A3	Y	21L858R	RET	R	41.7	multi.lung nodules	3/15	c.98C > G	3.32	Missense	p.S33C
5	64	F	RLL	Lobec.	1A3	N	19 Del	MLH1	R	14.0	Brain	3/15	c.121A > G	12.7	Missense	p.T41A
6	75	F	LUL	Lobec.	1Bv^b^	N	WT	No	R	33.3	multi.lung nodules	3/15	c.110C > T	12.1	Missense	p.S37F
7	76	F	RLL	Lobec.	1A2	N	WT	KIF5B-RET	R	60.3	RUL	3/15	c.100G > A	14.2	Missense	p.G34R
8	61	M	RML	Lobec.	IIB	Y	19 Del	TP53	R	8.5	Lt adrenal	3/15	c.98C > G	22.7	Missense	p.S33C
9	76	M	RUL	Lobec.	IIB	N	WT	PIK3CA,TP53, SMAD4	R	2.7	multi.lung nodules	9/15	c.1271T > G	6.5	Missense	p.L424R

Case	Age	Sex	Site	Surgery	Stage	Adjuvant	Fusion mutation	Concurrent alterations	Recurrence	RFS	Recurrence site	Validation methods of fusions
1	71	M	LLL	Lobec.	1Bv	Y	ALK	APC, MLH1	R	42.7	RML	FISH
2	47	M	RLL	Lobec.	1A2	N	ALK	None	R	33.5	Multiple	IHC
3	30	M	RML	Lobec.	1A3	N	ALK	None	R	42.8	Brain	FISH
4	64	F	LLL	Wedge	1A3	Y	ALK	None	R	14.0	4R LN	FISH
5	64	M	RLL	Lobec.	1Bv	Y	ALK	None	N	53.5	None	FISH
6	82	M	LUL	Lobec.	1A2	N	ALK	None	N	50.1	None	IHC
7	81	M	LUL	Lobec.	1Bv	N	ALK	None	R	11.1	Brain, lung	IHC
8	71	F	RLL	Lobec.	1A3	N	ROS1	21L858R	R	21.6	Brain	FISH
9	60	M	RUL	Wedge.	1A2	Y	ROS1	None	N	56.4	None	RT-PCR
10	53	F	LUL	Lobec.	IIB	Y	ROS1	None	R	30.5	Lung	RT-PCR
11	76	F	RLL	Lobec.	1A2	N	RET	CTNNB1	R	60.3	RUL, multiple bone	FISH
12	59	F	RLL	Lobec.	1Bs	v	RET	None	R	37.0	4R LN	FISH

*Adjuvant* Adjuvant chemotherapy, *mon* Months, *FISH* Fluorescent in situ hybridization, *F* Female, *Lobec* Lobectomy, *IHC* Immunohistochemistry, *Multi.* Multiple, *M* Male, *LN* Lymph node, *LUL* Left upper lobe, *LLL* Left lower love, *R* Recurrence, *N* No recurrence, *RUL* Right upper lobe, *RML* Right middle lobe, *RLL* Right lower lobe, RFS Recurrence-free survival, *19 Del* 19 Deletion, *NA* Not available, *WT* Wild type, *VAF* Variant allele frequency, *Wedge*. Wedge resection

^a^1Bs means the tumor size is 3–4 cm

^b^1Bv means the tumor invades viceral-pleura

The CTNNB1 and fusion mutations are the genetic biomarkers to predict recurrence, allowing patients with the mutation to treat Tyrosine Kinase Inhibitor (TKI) in time. Therefore, overall survival data and treatment outcomes of TKI after recurrence are necessary to measure the benefit of genetic information by NGS. However, most patients in our study refused further treatment due to old age or high TKI cost, and only 4 patients received TKI treatment after recurrence. The data to analyze the benefit are insufficient in our study, so larger studies will be required.

## Discussion

Mutation profiles of stage I–II lung adenocarcinoma were analyzed using targeted NGS with panels of 170 cancer-related genes and 37 fusion-related genes. To identify the most potent genomic alterations contributing to recurrence, we analyzed early-stage lung adenocarcinoma with low tumor burden. As a result, the CTNNB1 mutations or fusion genes were independent negative predictive factors in multivariate analysis despite the resected small size cancers. Relapse caused by CTNNB1 mutation or fusion genes accounted for approximately 30% of all recurrence cases of stage I lung adenocarcinoma.

In our study, EGFR mutations (52.2%) were the most frequent genetic alterations because of the prevalence of lung adenocarcinoma in Asia.^17^–^19^ Notably, the frequency of TP53 mutations (18.3%) was lower than that reported in previous studies (30–60%).^19^,^20^ The frequency of TP53 mutations increased as the stage increased.^8^ The reason for the low frequency of TP53 can be explained by our cohort of early-stage lung adenocarcinoma. The prevalence of KRAS (14.3%) was similar to that in previous studies.^19^

RFS was not significantly affected by the number of pathogenic mutations. Interestingly, patients without driver mutations (*n* = 23, 11.4%) showed as short RFS as those patients with multiple mutations (Supplement Fig. 2). The unknown genetic alterations, RNA editing factors mutations, transcription factor mutations, or epigenetic alterations, except known driver mutations, might cause recurrence in the tumors without alteration.^11^ The number of mutations in the targeted NGS did not seem to be affected by clinical factors such as age or smoking history. However, previous whole gene or whole exon sequencing studies showed an association with the number of mutations and clinical factors. This may be because the number of mutations may vary depending on the number of cancer-related genes in the NGS panel, the sequencing depth, or cutoff levels called actionable mutations. In our targeted NGS, the number of cancer-related genes in the NGS panel (200 genes) was fewer than that in previous studies in whole exon sequencing (more than 300 genes).

We analyzed the RFS of subgroups classified according to the patient groups with multiple pathogenic mutations (Fig. 3e). EGFR mutations have been shown to be good prognostic markers.^7^,^18^,^21^ KRAS or TP53 mutations have been reported as poor prognosis markers.^20^,^22^ In our study, the RFS of an EGFR mutation and EGFR/TP53 co-mutations did not show any difference in recurrence. Our results were in accordance with the LACE (Lung Adjuvant Cisplatin Evaluation)-Bio study.^23^ There was no significant difference in RFS among patients with KRAS mutations and KRAS wild-type tumors (Supplementary Fig. S4). However, RFS in patients with KRAS/TP53 co-mutation was shorter than that in patients with a single KRAS mutation. These observations were consistent with previous studies.^15^,^24^ We need to consider the combined effects of TP53 and KRAS mutations on recurrence when evaluating the outcome.

Mutations in the gene encoding β-catenin, CTNNB1 mutations, were related to recurrence of early-stage lung adenocarcinoma in our study. We examined the reasons why most of the patients with CTNNB1 mutations had recurrence despite complete tumor resection. Accumulating data suggest that the Wnt/β-catenin pathway is involved in tumorigenesis and metastasis of lung cancer. In the normal state, β-catenin is degraded by the destruction complex consisting of adenomatous polyposis coli (APC), axin, and glycogen synthase kinase 3b (GSK3b). If it is not degraded by the aberration of Wnt/β-catenin signaling, it remains in the cytoplasm.^25^ The increased β-catenin in the cytoplasm moves to the nucleus, acts as a transcription factor to activate cell cycles continuously, and induces tumor formation.^15^,^16^,^26^,^27^ In our study, relapses of resected tumors with CTNNB1 mutations usually occur in distant organs. Considering the reports that CTNNB1 mutations were related to metastasis, they were thought to be related to micrometastasis undetected on radiologic or pathologic examination at the time of surgery. Furthermore, CTNNB1 mutations were more frequently detected in EGFR mutant tumors than in EGFR wild-type tumors. In a previous study, Nakayama et al. reported that β-catenin contributed to EGFR mutant-tumor development.^16^

We analyzed the effect of adjuvant chemotherapy in patients with CTNNB1 mutations. Unfortunately, the patients with CTNNB1 mutations experienced recurrence irrespective of adjuvant chemotherapy (*p* = 0.51). Adjuvant chemotherapy for CTNNB1 mutation is not effective in preventing recurrence. CTNNB1s have been reported to be resistant to chemotherapy in previous research.^25^ Thus, patients with CTNNB1 mutations need intensive surveillance for the early detection of recurrence and targeted β-catenin pathway therapy in clinical trials should be the treatment for them.^28^

We also observed that fusion genes (ALK, ROS1, or RET) were related to shorter RFS. In previous reports, early ALK positivity of the tumor was associated with a poor outcome in lung adenocarcinoma.^29^–^31^ Therefore, it is important to treat with adjuvant-targeted therapy or pemetrexed-based adjuvant chemotherapy for resected early stage lung cancer with ALK or fusion mutation.^32^

Targeted NGS has many benefits for early lung adenocarcinoma. It provides rich genetic information with reduced cost and time compared with whole exome sequencing. It can detect not only targetable driver mutations but also rare or non-hotspot mutations to predict prognosis, such as CTNNB1 mutations and fusion genes. NGS information enables the enrollment of relapsed patients in ongoing clinical trials for new targeted therapy. Recently, there have been many targeted therapies based on NGS information. For example, MRTX849 and AMG510^33^ are approved by the FDA as KRAS G12C inhibitors; RET inhibitor, BLU-667, LOXO-292, and RXDX-105 are in clinical trials;^34^ and crizotinib and ceritinib are available as ALK/ROS inhibitors.^35^ In addition, NGS studies help to assess recurrence risk and to select treatment strategies accordingly. For example, a short-term follow-up and adjuvant-targeted therapy could be performed for patients in the high-risk group with CTNNB1 mutation or fusion genes.^36^,^37^

One of the strengths of our research is that our cohort is homogeneous and consisted of early-stage lung adenocarcinoma only, not including squamous cell carcinoma or advanced stage, which is optimal for identifying the most potent recurrence-related oncogenes. Second, our study has no physician selection bias because most of the early-stage lung adenocarcinomas at our institution during the past 12 years were included, and NGS sequencing was performed by an outside company (Macrogen Inc.), which has a well-established and validated NGS pipeline. Third, our patients had a long-term follow-up period of more than 4 years. Lastly, the NGS results are accurate because all samples are from surgical resection, not from small percutaneous needle biopsy.

Our study has some limitations. First, germline mutations were not excluded because we did not collect blood or normal tissue because of the retrospective nature of the study. To prevent reporting germline false positives, data were filtered using a large human database and the house database of Macrogen Inc. Second, we did not perform RNA-NGS sequencing to identify the fusion genes; however, the gene rearrangement results of DNA were confirmed by FISH, RT-PCR, or D5F3 IHC. Third, our results are not confirmed by using a large database such as TCGA, which includes other ethnicities, because TCGA contains data on survival but not recurrence. Fourth, the number of patients in the study was not large. However, 230 patients is not a small number for an NGS study focused only on early lung adenocarcinoma, compared with previous NGS studies.^11^,^27^ The CTNNB1 and fusion genes were rare, and the recurrence rate of early lung cancer was low, so the number of patients with recurrence-related specific genes was inevitably small. However, these genes showed strong correlations with recurrence. The aim of this research was to determine whether targeted NGS can identify recurrence-related genes and to identify molecular biomarkers for ambiguous stage IB risk stratification. The number of patients was sufficient to achieve this goal.

A finding in this NGS study was that CTNNB1 mutations or fusion genes were independent negative prognostic factors for recurrence in early-stage lung adenocarcinoma. A single EGFR mutation was a good prognostic marker, but EGFR/CTNNB1 co-mutations showed a significantly shorter RFS even for stage I adenocarcinoma, most of which were cured. We should consider the impact of concomitant mutations with a conventional driver mutation to predict recurrence risk. This study suggests that targeted NGS provides valuable information to predict recurrence and identify patients at high recurrence risk. In addition, targeted NGS helps to select the optimal treatment strategy among intensive surveillance and adjuvant-targeted therapy.

## Electronic Supplementary Material

Below is the link to the electronic supplementary material.

Supplementary material 1 (DOCX 206 kb)

## Data Availability

The datasets used and/or analyzed during the current study are available from the corresponding authors upon reasonable request.
