# Supplementary material for: Targeted Next-Generation Sequencing Analysis for Recurrence in Early-Stage Lung Adenocarcinoma
Source: Ann Surg Oncol. 2020 Nov 2;28(7):3983–93. doi: 10.1245/s10434-020-09276-x (PMC8184531; doi:10.1245/s10434-020-09276-x)
Supplement: Supplementary file 1 — Supplementary material 1 (DOCX 206 kb) [file 10434_2020_9276_MOESM1_ESM.docx]

**Supplementary figures**

**INDEX**

**Supplementary Figure S1. Frequency of genetic alterations in 230 whole lung adenocarcinomas**

**Supplementary Figure S2. Kaplan-Meier curve comparing the RFS according to the number of genetic alterations**

**Supplementary Figure S3. Kaplan-Meier curve comparing the RFS according to adjuvant chemotherapy in patients with CTNNB1 mutations**

**Supplementary Figure S4. Kaplan-Meier curve comparing the RFS according to KRAS mutation status.**

**Supplementary Table S1. List of 170 target genes and 37 fusion-related genes.** **(KF1 panel)**

**Supplementary method 1. NGS methods**

**Supplementary method 2. Validation of fusions**


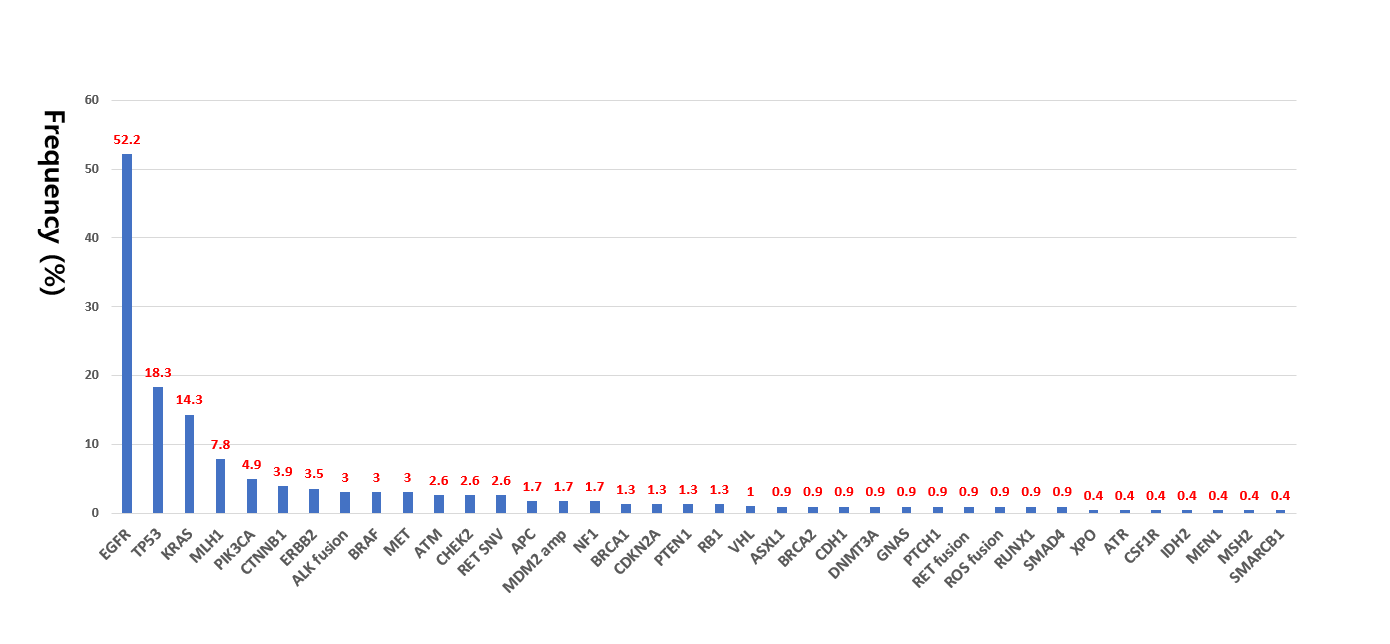


**Supplementary Figure S1. Frequency of genetic alterations in 230 stage I-II lung adenocarcinomas.**


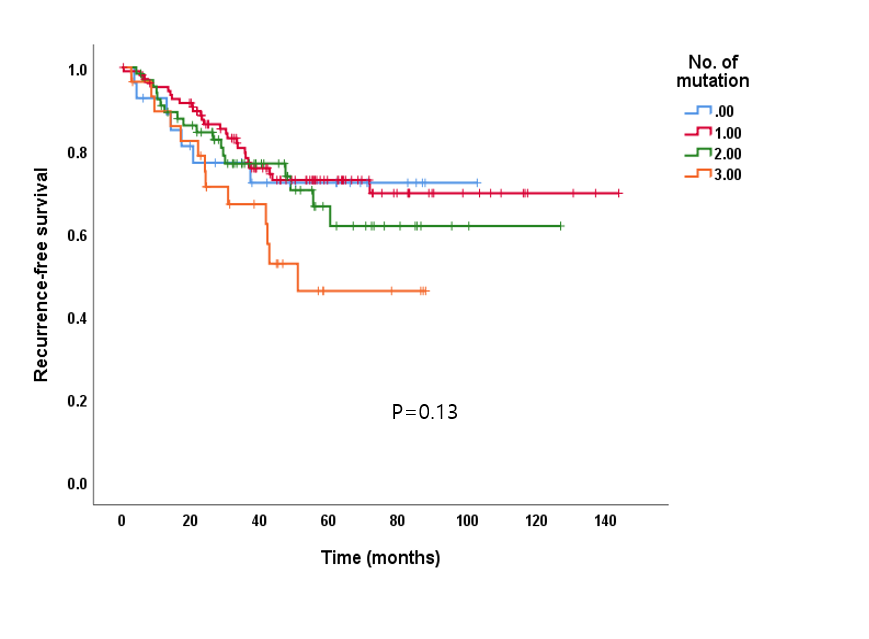


**Supplementary Figure S2. Kaplan-Meier curve comparing the RFS according to the number of genetic alterations.** The RFS according to the number of mutations showed no significant difference.


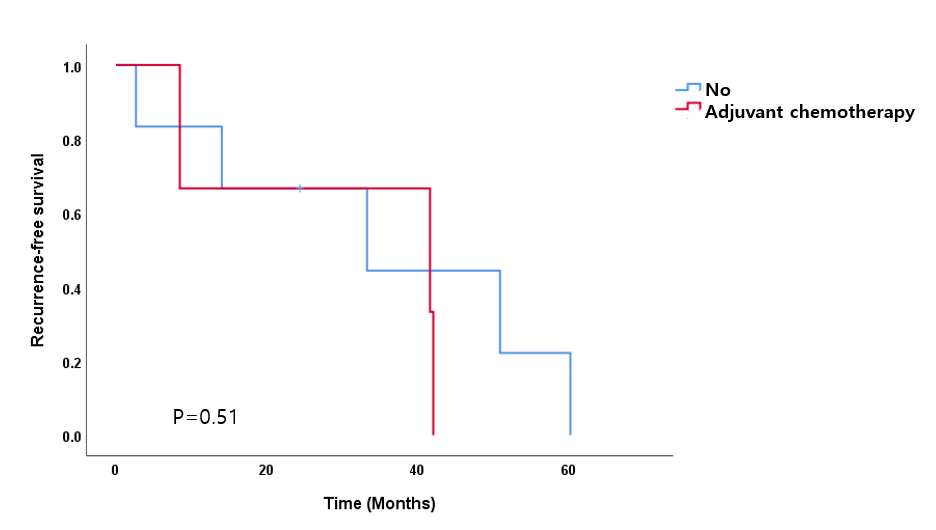


**Supplementary Figure S3 Kaplan-Meier curve comparing the RFS according to adjuvant chemotherapy in patients with CTNNB1 mutations.** There is no significant difference in RFS

**.**
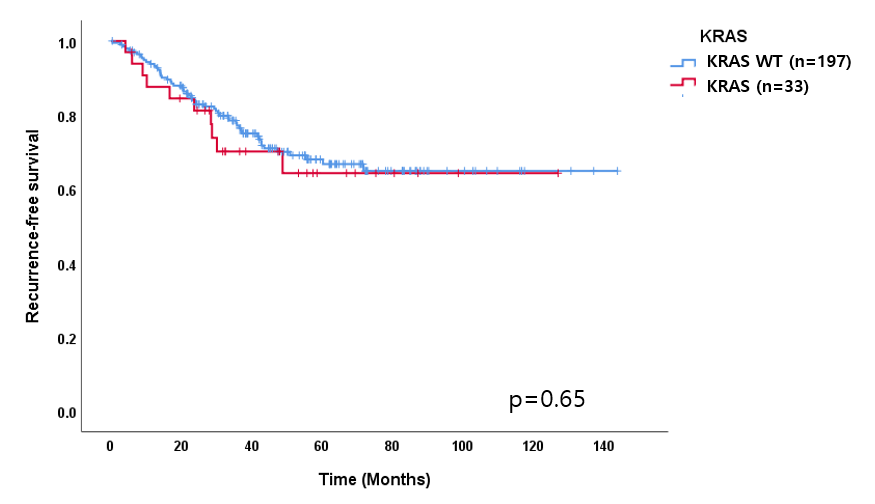


**Supplementary Figure S4. Kaplan-Meier curve comparing the RFS according to KRAS mutation status.** There is no significant difference in RFS.

**Supplementary table S1. List of 170 target genes and 37 fusion- related genes (KF1 panel)**

| Target Gene | Target Gene | Target Gene | Target Gene | Target Gene | Target Gene | Fusion gene | Fusion gene |
| --- | --- | --- | --- | --- | --- | --- | --- |
| ABL1 | CDH1 | ETV4 | KIT | NKX2-1 | RHEB | ABL1 | RET |
| ABL2 | CDK12 | ETV5 | KMT2A | NOTCH1 | RICTOR | AKT3 | ROS1 |
| AKT1 | CDK4 | ETV6 | KRAS | NOTCH2 | RNF43 | ALK | SS18 |
| AKT2 | CDK6 | EWSR1 | MAP2K1 | NOTCH3 | ROS1 | BCL2 | TCRVB |
| AKT3 | CDKN1A | EZH2 | MAP2K2 | NOTCH4 | RSPO1 | BCL6 | TMPRSS2 |
| ALK | CDKN1B | FBXW7 | MAP2K4 | NPM1 | RSPO2 | BRAF | TRAC |
| APC | CDKN2A | FGFR1 | MAP3K1 | NRAS | RUNX1 | CIITA | TRGC2 |
| AR | CDKN2B | FGFR2 | MAP3K4 | NTRK1 | SMAD2 | EGFR | TVP23A |
| ARAF | CDKN2C | FGFR3 | MAPK1 | NTRK2 | SMAD4 | ERG |  |
| ASXL1 | CEBPA | FGFR4 | MAPK3 | NTRK3 | SMARCA4 | ETV1 |  |
| ATM | CHEK2 | FLCN | MAPK8 | NUTM1 | SMARCB1 | EWSR1 |  |
| ATR | CREBBP | FLT1 | MCL1 | PDGFB | SMO | FAM30A |  |
| AURKA | CRKL | FLT3 | MDM2 | PDGFRA | SRC | FGFR1 |  |
| AURKB | CSF1R | FLT4 | MDM4 | PDGFRB | STK11 | FGFR2 |  |
| AURKC | CTNNB1 | FOXL2 | MED12 | PIK3CA | SYK | FGFR3 |  |
| AXL | DDR1 | GNA11 | MEN1 | PIK3CB | TET2 | FUS |  |
| BAP1 | DDR2 | GNAQ | MET | PIK3CD | TMPRSS2 | IGHE |  |
| BCL2 | DNMT3A | GNAS | MITF | PIK3R1 | TOP2A | IGLL5 |  |
| BRAF | DOT1L | HDAC9 | MLH1 | PIK3R2 | TP53 | JAK2 |  |
| BRCA1 | EGFR | HGF | MPL | POLE | TSC1 | KMT2A |  |
| BRCA2 | EPHA3 | HRAS | MSH2 | PPARG | TSC2 | MYC |  |
| BRD2 | ERBB2 | IDH1 | MSH6 | PTCH1 | VHL | NPM1 |  |
| BRD3 | ERBB3 | IDH2 | MTOR | PTEN | WT1 | NTRK1 |  |
| BRD4 | ERBB4 | IGF1R | MYC | RAB35 | XPO1 | PAX5 |  |
| CBFB | ERCC2 | IGF2 | MYCN | RAD50 | ZNRF3 | PDGFRA |  |
| CCND1 | ERG | JAK1 | MYD88 | RAF1 |  | PDGFRB |  |
| CCND2 | ERRFI1 | JAK2 | NF1 | RARA |  | PPARG |  |
| CCND3 | ESR1 | JAK3 | NF2 | RB1 |  | RAF1 |  |
| CCNE1 | ETV1 | KDR | NFKBIA | RET |  | RARA |  |

**Supplementary method 1. NGS methods**

Adapter sequences were removed by Cutadapt (v1.9.1)^1^. Fast QC (v0.11.5) software was used for FASTQ file quality control. Sequencing reads were mapped to the human genome version 19 (hg19) using the Burrows-Wheeler Aligner (BWA-mem, v0.7.12)^2^. Poorly mapped reads (mapping quality <20) were removed using Samtools (v.1.3.1)^3^. Local realignment around indels and base quality score recalibration were performed with the Genome Analysis Toolkit (GATK 3.4) (http://www.broadinstitute.org/gatk/download). Duplicated reads were discarded using Picard Mark Duplicates (version 2.2.4)(https://software.broadinstitute.org/gatk/documentation/tooldocs). Somatic mutations, including single nucleotide variants (SNVs) and small insertions and deletions (indels), were identified using the MuTect2 algorithm^4^. False-positive variant calls originating from oxoG artifacts were then excluded. Mutations below 2% variant allele frequency were also excluded. All variants were annotated using SnpEff and SnpSift v4.3i ^5^ with dbNSFP v2.9.3^6^. Fusion genes were identified using an in-house script that can discriminate plausible gene fusion events among the structural variations predicted by LUMPY 0.2.13^7^. Copy number variations of the target genes were detected using MuTect2 with an in-house copy number caller. The fusion reads were further manually reviewed and confirmed in Integrative Genomics Viewer(https://software.broadinstitute.org/software/igv/download). Variant calls were further analyzed using the COSMIC database(http://cancer.sanger.ac.kr), dbSNP build 142, and amino acid change information. False-positive variants were excluded because they were found in misalignments and were filtered out by using the in-house normal population variant database KPGP. We removed synonymous mutations and mutations of unknown significance and included only non-synonymous SNVs and indels in coding exons and splicing sites as the pathogenic mutations. We included pathogenic mutations with a functional impact on the tumor. According to the ACMG/AMP(https://www.acmg.net/docs/Standards_Guidelines guidelines), only tier 1 and tier 2 variants were analyzed. Details of the target genes of the KF1 panel (170 genes) and 37 fusion-related genes are available in Supplement table S1. A threshold of 10 copies was used for gene amplification. In case of fusion genes, we validated the results by anaplastic lymphoma kinase (ALK) immunohistochemistry (D5F3 clone) or ALK fluorescent in situ hybridization (FISH) using the Vysis break-apart probe (Abbott Molecular, Abbott Park, IL, USA) for ALK rearrangement, quantitative RT-PCR (Amoy) for ROS arrangement and FISH for RET arrangement.

**Supplementary method 2. Validation of fusions**

RET FISH tests were performed on formalin-fixed paraffin-embedded (FFPE) tumor tissues using SPEC RET Dual Color Break Apart Probes according to the manufacturer’s instructions (ZytoVision, Bremerhaven, Germany). The SPEC RET Dual Color Break Apart Probe is a mixture of two direct labeled probes hybridizing to the 10q11.21 band. The orange fluorochrome direct labeled probe hybridizes proximal to the RET gene, the green fluorochrome direct labeled probe hybridizes distal to the gene.

*ROS1* fusion was detected by using an AmoyDx® *ROS1* fusion gene detection kit (Amoy Diagnostics). The quantity and quality of RNA was subsequently determined on a NanoDrop 2000 Spectrophotometer (Thermo Fisher Scientific, Waltham, MA, USA). mRNA extracted from cytological specimens and tumor tissue was reverse transcribed to cDNA at 42°C, and then amplified by PCR. The RT-PCR conditions were as follows: 95°C for 5 minutes, 15 cycles of denaturation at 95°C for 25 seconds, annealing at 64°C for 20 seconds, and elongation at 72°C for 20 seconds to ensure specificity, and then up to 31 cycles at 93°C for 25 seconds, 60°C for 35 seconds (data collection), and 72°C for 20 seconds.

**References**

1. Martin M. Cutadapt removes adapter sequences from high-throughput sequencing reads. *EMBnet.* 2011 2011;17:3 J

2. Li HJapa. Aligning sequence reads, clone sequences and assembly contigs with BWA-MEM. *arXiv* 2013.

3. Li H, Handsaker B, Wysoker A, et al. The sequence alignment/map format and SAMtools. *Bioinformatics* 2009; 25:2078-2079.

4. Cibulskis K, Lawrence MS, Carter SL, et al. Sensitive detection of somatic point mutations in impure and heterogeneous cancer samples. *Nat biotechnol* 2013; 31:213.

5. Cingolani P, Platts A, Wang LL, et al. A program for annotating and predicting the effects of single nucleotide polymorphisms, SnpEff: SNPs in the genome of Drosophila melanogaster strain w1118; iso-2; iso-3. *Fly* 2012; 6:80-92.

6. Liu X, Wu C, Li C, et al. dbNSFP v3. 0: A one‐stop database of functional predictions and annotations for human nonsynonymous and splice‐site SNVs. *Hum mutant.* 2016; 37:235-241.

7. Layer RM, Chiang C, Quinlan AR, et al. LUMPY: a probabilistic framework for structural variant discovery. *Genome Biology* 2014; 15: R84.

**Supplementary method 3. Validation of fusions**

RET FISH tests were performed on formalin-fixed paraffin-embedded (FFPE) tumor tissues using SPEC RET Dual Color Break Apart Probes according to the manufacturer’s instructions (ZytoVision, Bremerhaven, Germany). The SPEC RET Dual Color Break Apart Probe is a mixture of two direct labeled probes hybridizing to the 10q11.21 band. The orange fluorochrome direct labeled probe hybridizes proximal to the RET gene, the green fluorochrome direct labeled probe hybridizes distal to the gene.

*ROS1* fusion was detected by using an AmoyDx® *ROS1* fusion gene detection kit (Amoy Diagnostics). The quantity and quality of RNA was subsequently determined on a NanoDrop 2000 Spectrophotometer (Thermo Fisher Scientific, Waltham, MA, USA). mRNA extracted from cytological specimens and tumor tissue was reverse transcribed to cDNA at 42°C, and then amplified by PCR. The RT-PCR conditions were as follows: 95°C for 5 minutes, 15 cycles of denaturation at 95°C for 25 seconds, annealing at 64°C for 20 seconds, and elongation at 72°C for 20 seconds to ensure specificity, and then up to 31 cycles at 93°C for 25 seconds, 60°C for 35 seconds (data collection), and 72°C for 20 seconds.

**References**

1. Martin M. Cutadapt removes adapter sequences from high-throughput sequencing reads. *EMBnet.* 2011 2011;17:3 J

2. Li HJapa. Aligning sequence reads, clone sequences and assembly contigs with BWA-MEM. *arXiv* 2013.

3. Li H, Handsaker B, Wysoker A, et al. The sequence alignment/map format and SAMtools. *Bioinformatics* 2009; 25:2078-2079.

4. Cibulskis K, Lawrence MS, Carter SL, et al. Sensitive detection of somatic point mutations in impure and heterogeneous cancer samples. *Nat biotechnol* 2013; 31:213.

5. Cingolani P, Platts A, Wang LL, et al. A program for annotating and predicting the effects of single nucleotide polymorphisms, SnpEff: SNPs in the genome of Drosophila melanogaster strain w1118; iso-2; iso-3. *Fly* 2012; 6:80-92.

6. Liu X, Wu C, Li C, et al. dbNSFP v3. 0: A one‐stop database of functional predictions and annotations for human nonsynonymous and splice‐site SNVs. *Hum mutant.* 2016; 37:235-241.

7. Layer RM, Chiang C, Quinlan AR, et al. LUMPY: a probabilistic framework for structural variant discovery. *Genome Biology* 2014; 15: R84.
